# Electrospray Ionization Mass Spectrometry of Apolipoprotein CIII to Evaluate *O*-glycan Site Occupancy and Sialylation in Congenital Disorders of Glycosylation

**DOI:** 10.5702/massspectrometry.A0104

**Published:** 2022-08-10

**Authors:** Yoshinao Wada, Nobuhiko Okamoto

**Affiliations:** 1 Department of Obstetric Medicine, Osaka Women’s and Children’s Hospital (OWCH), 840 Murodo-cho, Izumi, Osaka 594–1101, Japan; 2 Department of Molecular Medicine, Osaka Women’s and Children’s Hospital (OWCH), 840 Murodo-cho, Izumi, Osaka 594–1101, Japan; 3 Department of Medical Genetics, Osaka Women’s and Children’s Hospital (OWCH), 840 Murodo-cho, Izumi, Osaka 594–1101, Japan

**Keywords:** congenital disorder of glycosylation, electrospray ionization mass spectrometry, *O*-glycosylation, apolipoprotein CIII, declustering potential

## Abstract

Congenital disorders of glycosylation (CDG) are inherited metabolic diseases that affect the synthesis of glycoconjugates. Defects in mucin-type *O*-glycosylation occur independently or in combination with *N*-glycosylation disorders, and the profiling of the *O*-glycans of apolipoprotein CIII (apoCIII) by mass spectrometry (MS) can be used to support a diagnosis. The biomarkers are site occupancy and sialylation levels, which are indicated by the content of non-glycosylated apoCIII0a isoform and by the ratio of monosialylated apoCIII1 to disialylated apoCIII2 isoforms, respectively. In this report, electrospray ionization (ESI) quadrupole MS of apoCIII was used to identify these biomarkers. Among the instrumental parameters, the declustering potential (DP) induced the fragmentation of the *O*-glycan moiety including the Thr–GalNAc linkage, resulting in an increase in apoCIII0a ions. This incurs the risk of creating a false positive for reduced site occupancy. The apoCIII1/apoCIII2 ratio was substantially unchanged despite some dissociation of sialic acids. Therefore, appropriate DP settings are especially important when transferrin, which requires a higher DP, for *N*-glycosylation disorders is analyzed simultaneously with apoCIII in a single ESI MS measurement. Finally, a reference range of diagnostic biomarkers and mass spectra of apoCIII obtained from patients with SLC35A1-, TRAPPC11-, and ATP6V0A2-CDG are presented.

## INTRODUCTION

Congenital disorder(s) of glycosylation (CDG) represent an umbrella of genetic diseases caused by mutations in genes related to the formation of glycoconjugates.^1,2)^ Among the major classes of glycoconjugates, a glycoprotein contains one or more oligosaccharides (glycans) that are covalently linked to a peptide backbone usually *via* *N*- or *O*-linkages. An *N*-glycan (*N*-linked oligosaccharide) is formed by enzymatic processes in the endoplasmic reticulum and the Golgi apparatus, and defects in *N*-glycosylation make up the largest subgroup of CDG. A diagnosis of congenital disorders of *N*-glycosylation is typically made by isoelectric focusing (IEF) or mass spectrometry (MS) of transferrin in combination with a genetic analysis.^3–6)^

In *O*-glycosylation, a sugar molecule is attached to the hydroxyl group of a serine or threonine residue in a protein molecule. The mucin type core-1 *O*-glycan is formed by an extension of oligosaccharides from an *N*-acetylgalactosamine (GalNAc) and is analyzed for CDG by using the IEF of apolipoprotein CIII (apoCIII), which contains only one *O*-glycan and no *N*-glycans.^7)^ The attachment of GalNAc to the apoCIII polypeptide is catalyzed by polypeptide *N*-acetylgalactosaminyltransferase 2, and a deficiency of this gene results in the loss of *O*-glycosylation of apoCIII in GALNT2-CDG.^8)^ Another type of abnormal *O*-glycan profile is found in combination with *N*-glycosylation disorders, because the *N*- and *O*-glycosylation systems share a portion of their pathways to produce the donor nucleotide sugars and depend on Golgi homeostasis. In particular, sialylation is often affected by structural or functional abnormalities in the Golgi apparatus. For example, defects in the component molecules in the conserved oligomeric Golgi (COG) complex affect membrane trafficking and Golgi homeostasis, thus causing a decreased level of sialylation in both *N*- and *O*-glycans.^1,9–11)^ However, in some cases, hyposialylation is observed in the *O*-glycans but not in *N*-glycans, emphasizing the importance of the analysis of *O*-glycans.^12)^

In 2012, matrix-assisted laser desorption/ionization (MALDI) MS enabled rapid analysis without the prior separation of apoCIII from serum was reported.^13,14)^ MS is superior to IEF in that it distinguishes non-glycosylated apoCIII isoforms from those containing neutral glycans.^15,16)^ An increase in the levels of non-glycosylated isoforms indicates a decrease in site occupancy and is typically observed in GALNT2-CDG.^8,17)^ Regarding sialylation, MALDI MS underestimates the sialylation levels due to in-source decay resulting in a significant loss of sialic acids during the ionization process.^13,15)^ Compared with MALDI, the loss of sialic acid from non-derivatized glycoconjugates is low in ESI,^18)^ and ESI MS is therefore widely used for the screening of congenital disorders of *N*-glycosylation. Considering that there are few reports on the ESI MS of apoCIII,^8,17)^ the technical aspects of the ESI MS of apoCIII using a quadrupole instrument to facilitate the screening of *O*-glycosylation disorders in clinical laboratories is reported here.

## MATERIALS AND METHODS

### Subjects and ethical approval

Sera without personally identifiable information were delivered from physicians who were in charge of the patients to Osaka Women’s and Children’s Hospital (OWCH) for the diagnosis of CDG. They were affected by developmental delay or multisystem diseases of unknown etiologies. This study was approved by the institutional review board of OWCH.

### Immunopurification of apoCIII

Immunopurification of apoCIII was performed in the same manner as was previously reported for transferrin.^19)^ An affinity column was prepared using a goat polyclonal antibody against human apoCIII (Academy Bio-Medical Co., Houston, TX, USA) and a ligand-coupling Sepharose cartridge (HiTrap NHS-activated HP 1 mL bed volume, GE Healthcare, Piscataway, NJ, USA). The antibody-coupled Sepharose was then recovered from the cartridge. A ten μL portion of serum was mixed with a 20-μL slurry of the antibody-coupled Sepharose in 0.5 mL of phosphate-buffered saline (PBS), and the solution was incubated at 4°C for 30 min. After washing with PBS, the apoCIII was eluted from Sepharose with 0.1 M glycine–HCl buffer at pH 2.5.

### Mass spectrometry

Liquid chromatography MS was conducted on an API4500 ESI-triple quadrupole mass spectrometer (Sciex, Framingham, MA, USA) connected to a C8 reversed phase column (2 mm diameter and 10 mm length, GL Sciences, Tokyo, Japan). After injection, the column was washed with 0.1% formic acid at a flow rate of 0.2 mL/min, and then eluted with 60% acetonitrile/0.1% formic acid at a flow rate of 0.05 mL/min.

The API4500 mass spectrometer was operated in the positive Q1 MS mode with the parameters as follows: gas temperature was at 120°C, curtain gas pressure was 10 psi, ion source gas pressure was 16 psi, IonSpray voltage was 5.5 kV, declustering, or orifice-skimmer, potential (DP) was 100 V (50–150 V), and entrance potential was 10 V. The full scan range was set from *m*/*z* 790 to 1650, and scan rate was 200 Da/s. Tandem mass spectrometry of *m*/*z* 1388.3 ions was performed with collision energies of 50 V. Mass calibration was conducted using polypropylene glycol. The zero-charge mass spectrum was generated by the Promass protein deconvolution software (Thermo-Fisher Scientific, Waltham, MA, USA).

### Statistical analyses

Statistical analyses were performed by using JMP statistical analysis software (SAS Institute, NC, USA). Multiple comparisons were performed by Tukey’s method.

## RESULTS AND DISCUSSION

### Isoform profiling by ESI MS

**Figure 1:**
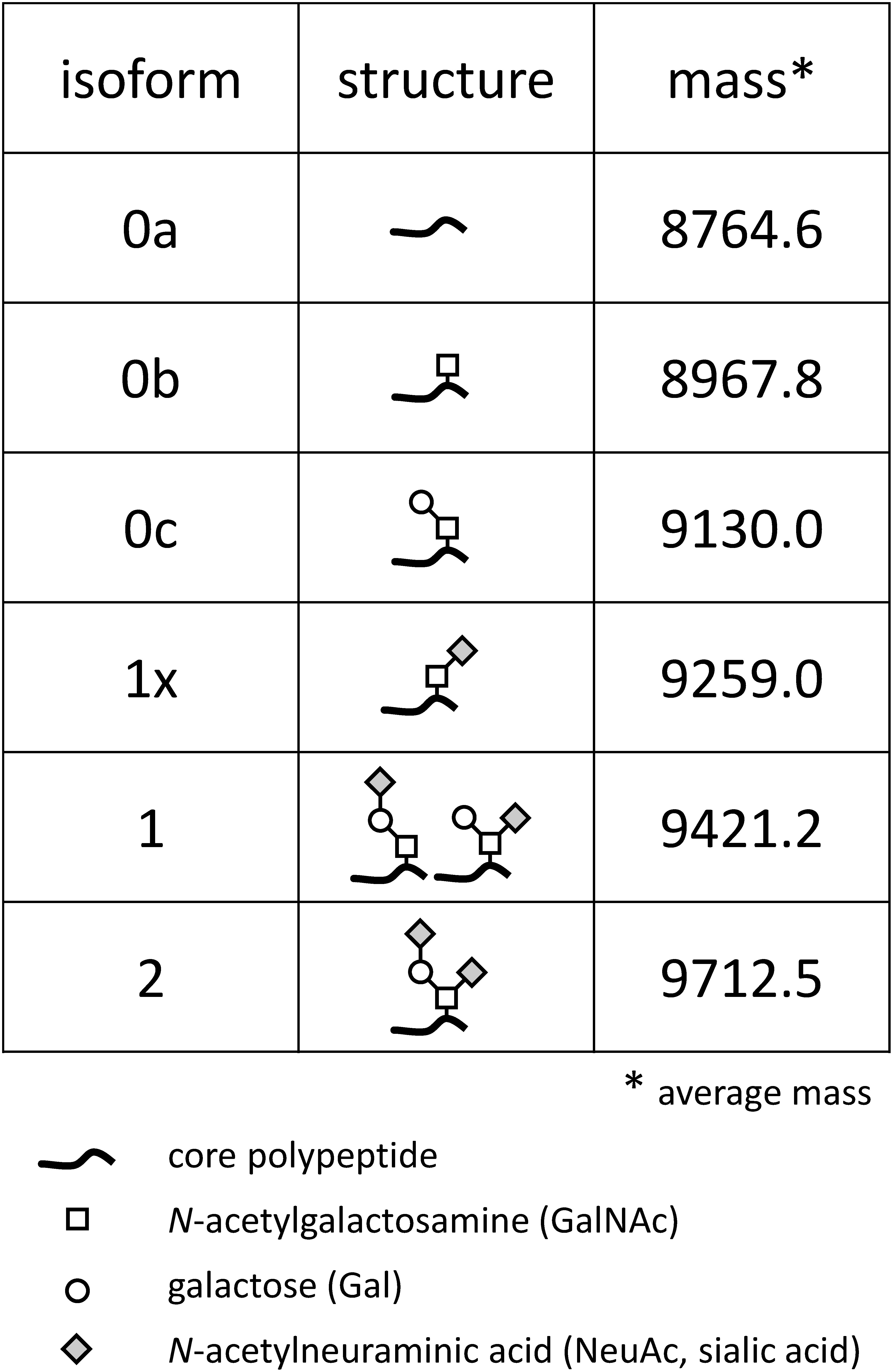
Fig. 1. Structure and molecular masses of apoCIII isoforms.

**Figure 2:**
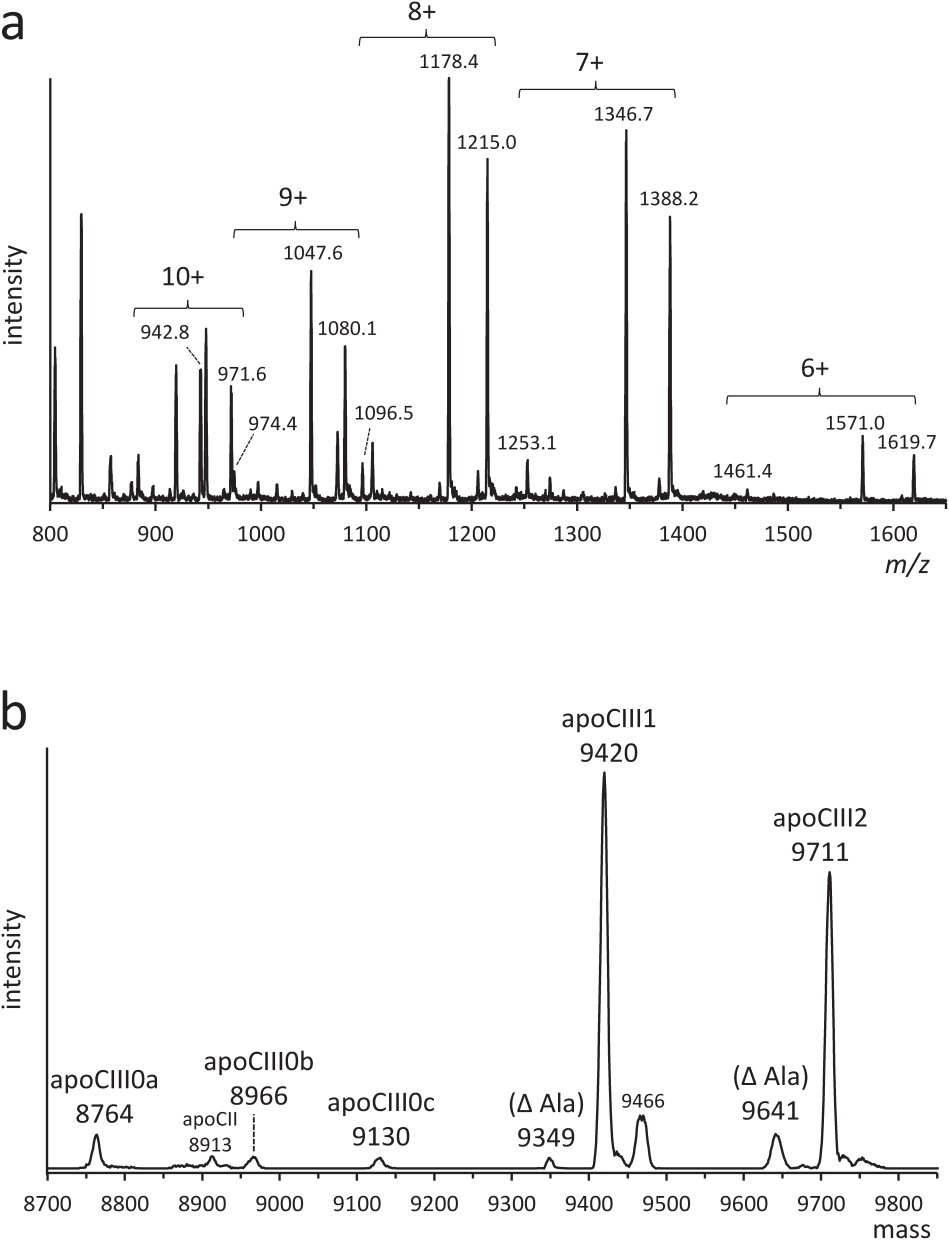
Fig. 2. ESI mass spectra of apoCIII from an individual with normal *O*-glycosylation. (a) Multiply-charged ion mass spectrum. Charge state is indicated above each isoform. (b) Deconvoluted mass spectrum. The spectrum data files are available in J-STAGE Data. https://doi.org/10.50893/data.massspectrometry.20076416

Human apoCIII contains a single mucin-type *O*-glycan attached to a threonine residue near the C-terminal end of the molecule. The apoCIII *O*-glycan is small, containing up to 4 saccharides (GalNAcGalNeuAc_2_). ApoCIII isoforms with different glycoforms, GalNAc, GalNAcGal, GalNAcGalNeuAc, GalNAcGalNeuAc_2_, are called apoCIII0b, 0c, 1, 2, respectively, and apoCIII0a is the non-glycosylated isoform (Fig. 1). The GalNAcGalNeuAc is a mixture of two isomers in which NeuAc is bound to GalNAc *via* an α2–6 linkage or to terminal Gal *via* an α2–3 linkage. The theoretical mass of these isoforms ranges from 8764.6 (apoCIII0a) to 9712.5 (apoCIII2), and ESI generates 6 to 10-charged ions observed in the mass spectrum range of *m*/*z* 800–1650. The major species are monosialylated (apoCIII1) and disialylated (apoCIII2) isoforms, and non-glycosylated apoCIII0a is present in most individuals. The charge state distributions of apoCIII1 and 2 are similar to each other, and the relative intensities of these isoforms are conserved for the same charge state ion pairs (Fig. 2a). Molecules containing neutral (oligo)saccharides, apoCIII0b and 0c, are observed in very small quantities (Fig. 2b). In addition, there are small peaks corresponding to molecules that lack the C-terminal alanine,^15)^ the abundance of which usually varies significantly between samples. Species with other modifications such as fucosylation^20)^ or carbamylation^21)^ were not observed in our samples. A small amount of apolipoprotein CII was co-immunopurified with apoCIII.

### Diagnostic isoforms

ApoCIII0a, 1 and 2 are key isoforms that are used for the diagnosis of CDG, and no disease in which apoCIII0b or 0c is specifically increased has been reported to date. Two major diagnostic biomarkers are a decrease in *O*-glycan occupancy and a decrease in sialylation levels. In IEF, site occupancy is evaluated by the density of the apoCIII0 band which is composed of apoCIII0a, b and c, and these “neutral” species can be separated by two-dimensional electrophoresis.^7,22)^ The decreased sialylation is estimated by the increased density of the apoCIII1 (and 0) band.^22)^ In MS, these biomarkers are evaluated based on the intensity of the corresponding ion peaks. MS may underestimate the sialylation level due to its charge-dependent effect on ionization efficiency and to fragmentation during the ionization process.^13,15)^ In ESI MS, the loss of sialic acid residues by fragmentation is much less pronounced compared with MALDI MS (Supplementary Figure S1).

### Evaluation of diagnostic markers

ESI MS or MALDI MS enables the label-free quantitation of sugar composition and site occupancy by calculating the signal intensity of ions in the mass spectrum.^23,24)^ This semi-quantitative method helps in comparing the profiles and site occupancy of *N*- and *O*-glycans of immunoglobulins between different samples and pathologies.^24,25)^ For apoCIII, site occupancy is calculated by the content of apoCIII0a ions in all apoCIII isoform ions as follows:
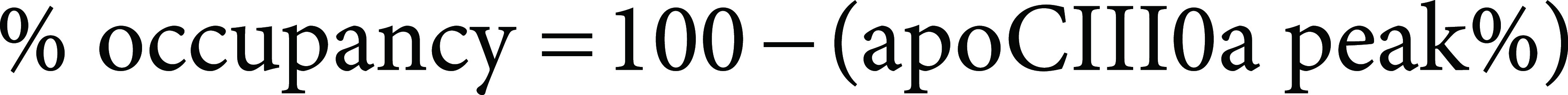
 where 
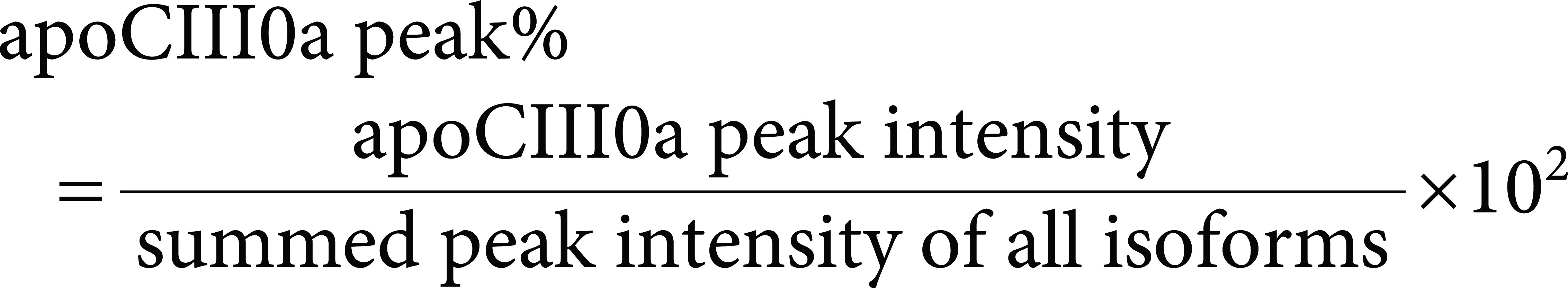


Sialylation level may be expressed by the molar content of sialic acid which is calculated based on the number of sialic acid residues of sialylated isoforms as follows.
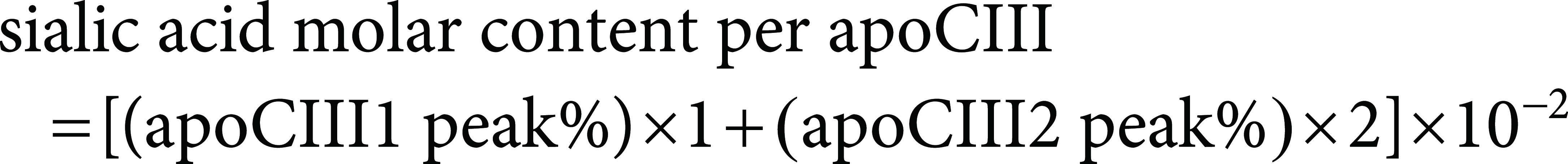



However, sialic acid content is not an independent diagnostic marker, since both the apoCIII1 peak% and apoCIII2 peak% are affected by another diagnostic marker, apoCIII0a peak%. Therefore, it is more appropriate to assess sialylation based on the ratio of apoCIII1 and 2.^14,15)^

### Effect of declustering potential

ESI requires DP, which is a voltage applied to the orifice plate. It is needed to prevent ions from forming clusters. DP causes collisional processes, resulting in better declustering of lower charge state ions and the fragmentation of higher charge state ions.^26)^ DP adjustment is required in order to achieve the optimum signal-to-noise ratio for the compound of interest, but increasing the DP also causes fragmentation, a process that is referred to as nozzle-skimmer dissociation.

**Figure 3:**
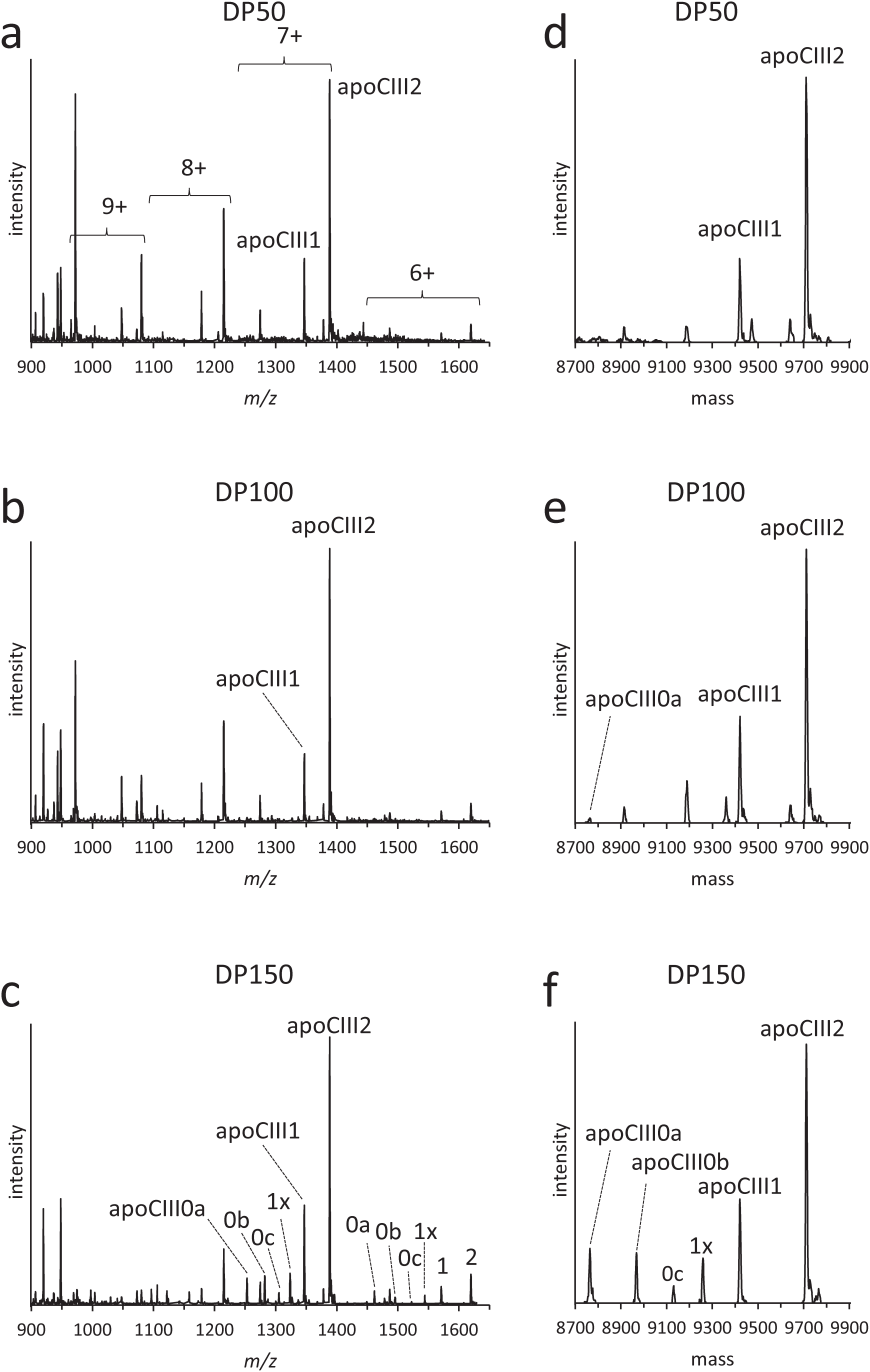
Fig. 3. ESI mass spectra at different DP values. A deconvoluted mass spectrum is shown on the right of each ESI mass spectrum. Various glycan-truncated forms including apoCIII1x are observed at DP150. The spectrum data files are available in J-STAGE Data. https://doi.org/10.50893/data.massspectrometry.20076434

**Figure 4:**
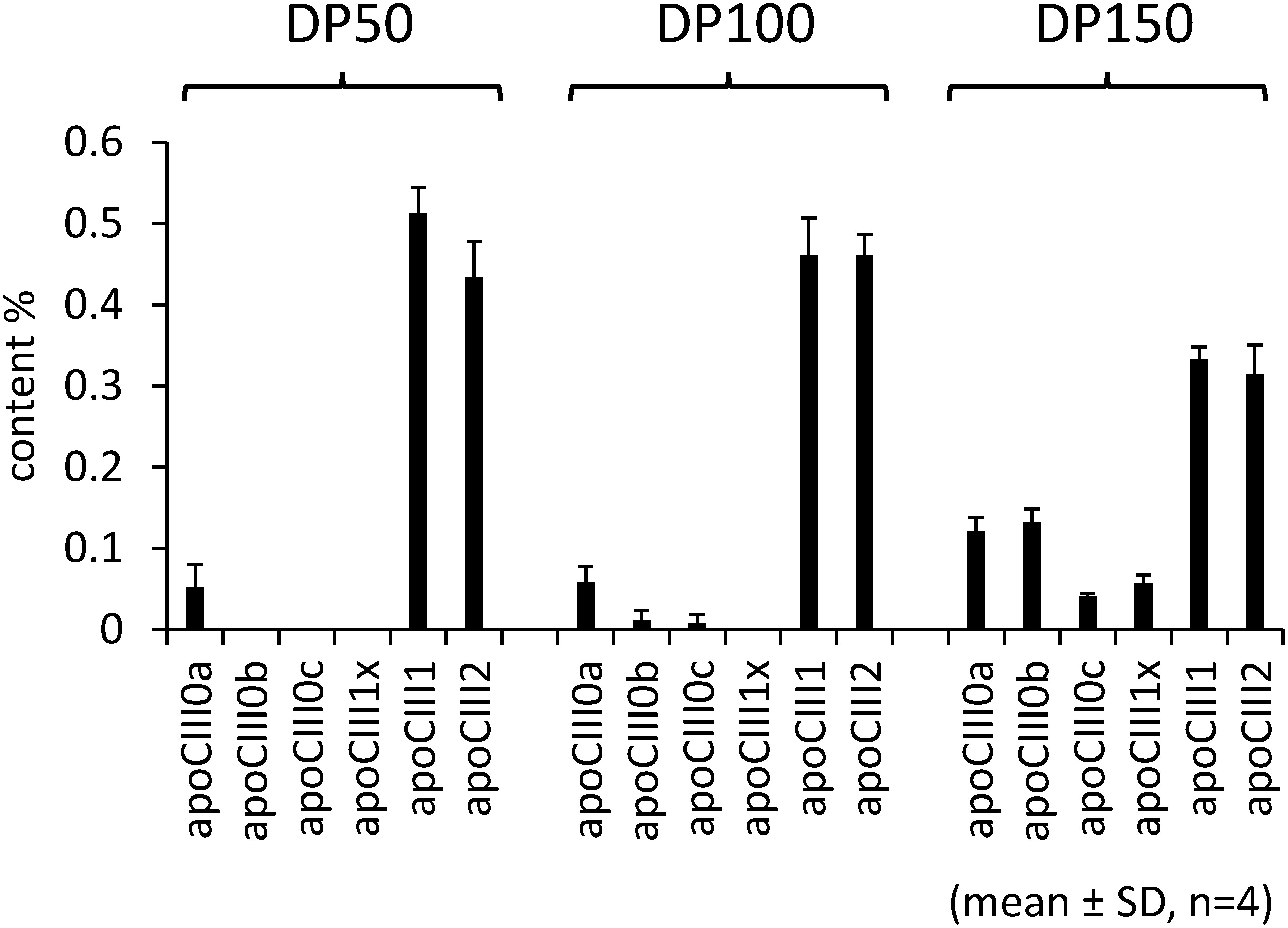
Fig. 4. Effect of DP on the levels of different apoCIII isoforms (*n*=4). Samples from four individuals with similar apoCIII1 and apoCIII2 contents were analyzed. Non-glycosylated isoforms and apoCIII1x significantly increased at DP150 (*P*<0.05), but the apoCIII1/apoCIII2 ratio was unchanged (*P*>0.05). Turkey’s multiple comparisons.

The effect of DP on the ESI mass spectrum of apoCIII was investigated using a highly sialylated apoCIII sample (Fig. 3). As the DP was increased from 50 V to 150 V, the charge state distribution shifted slightly to the high mass region. At DP150, the desialylated isoforms (apoCIII0a, 0b and 0c) appeared, and the molar content of sialic acid was reduced by 30% from DP50 or DP100. However, the apoCIII1/apoCIII2 ratio was not dependent on the DP values, probably due to a balance between the conversion from apoCIII2 to apoCIII1 and from apoCIII1 to the apoCIII0 isoforms. On the other hand, the apoCIII0a as well as other truncated species including apoCIII1x increased due to the fragmentation at DP150. These results were confirmed in samples in which the intensities of apoCIII1 and apoCIII2 ions were similar to each other (Fig. 4).

**Figure 5:**
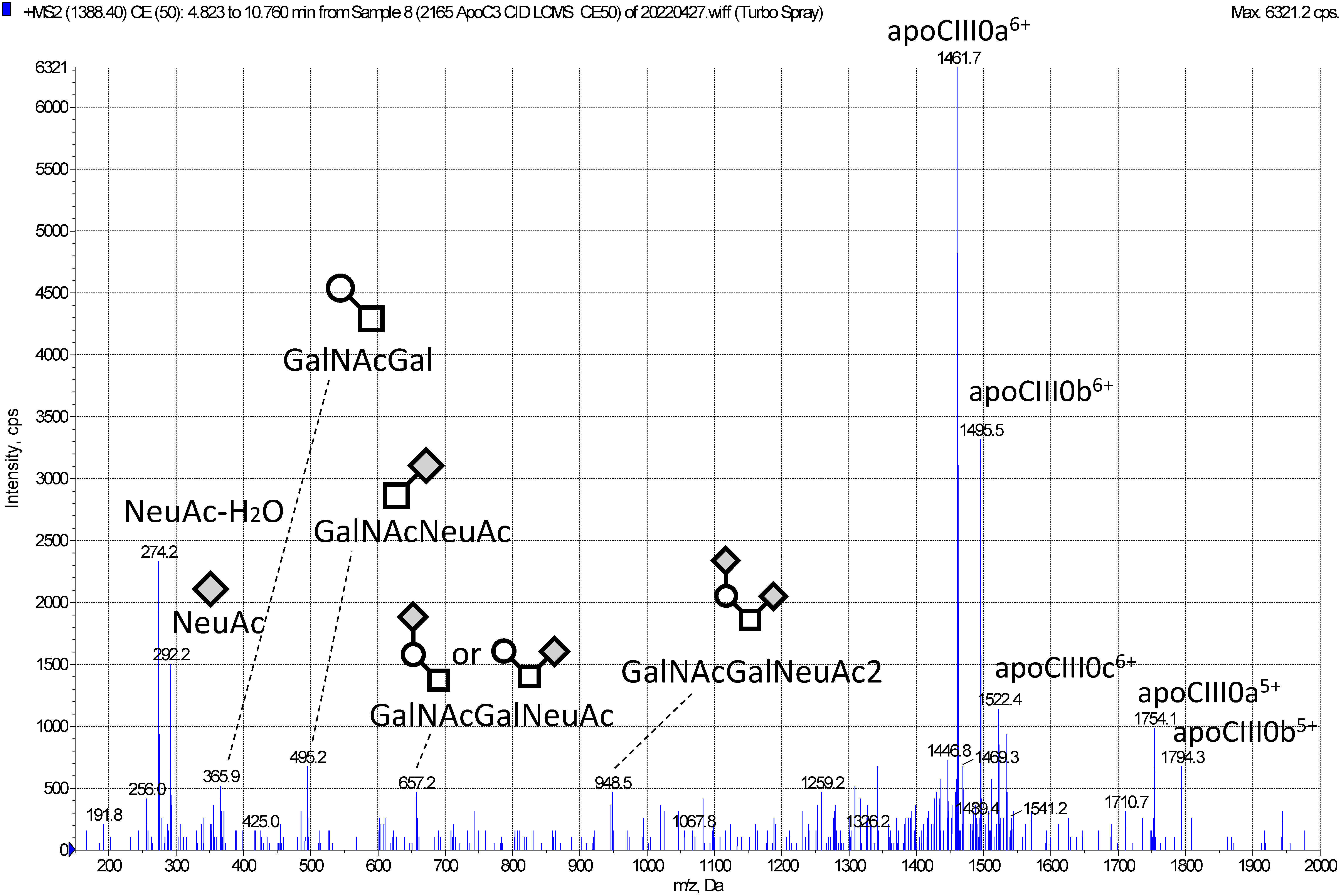
Fig. 5. MS/MS spectrum of [apoCIII2 + 7H]^7+^ ions activated by 50 V collision energy (CE). Various product ions derived from the dissociation of the glycan moiety were observed. See the supplementary file for MS/MS spectrum at CE40 V, where precursor ions remained. The spectrum data files are available in J-STAGE Data. https://doi.org/10.50893/data.massspectrometry.20076443

To further investigate the dissociation of the Thr–GalNAc bond, tandem MS was performed on the apoCIII2 ion. Collision activation produced apoCIII0 ions by the cleavage of Thr–GalNAc as well as a series of sugar fragments, reproducing the fragmentation caused by DP (Fig. 5 and Supplementary Figure S2).

These findings indicate that attention should be paid to the occupancy represented by apoCIII0a rather than the sialylation level when setting the DP. The DP issue raises concerns about the simultaneous measurement of transferrin and apoCIII, because the DP increases with increasing molecular mass of the target molecule and transferrin (80 kDa) requires a higher DP than apoCIII.^17,19)^

### Statistical evaluation and patients with CDG

**Figure 6:**
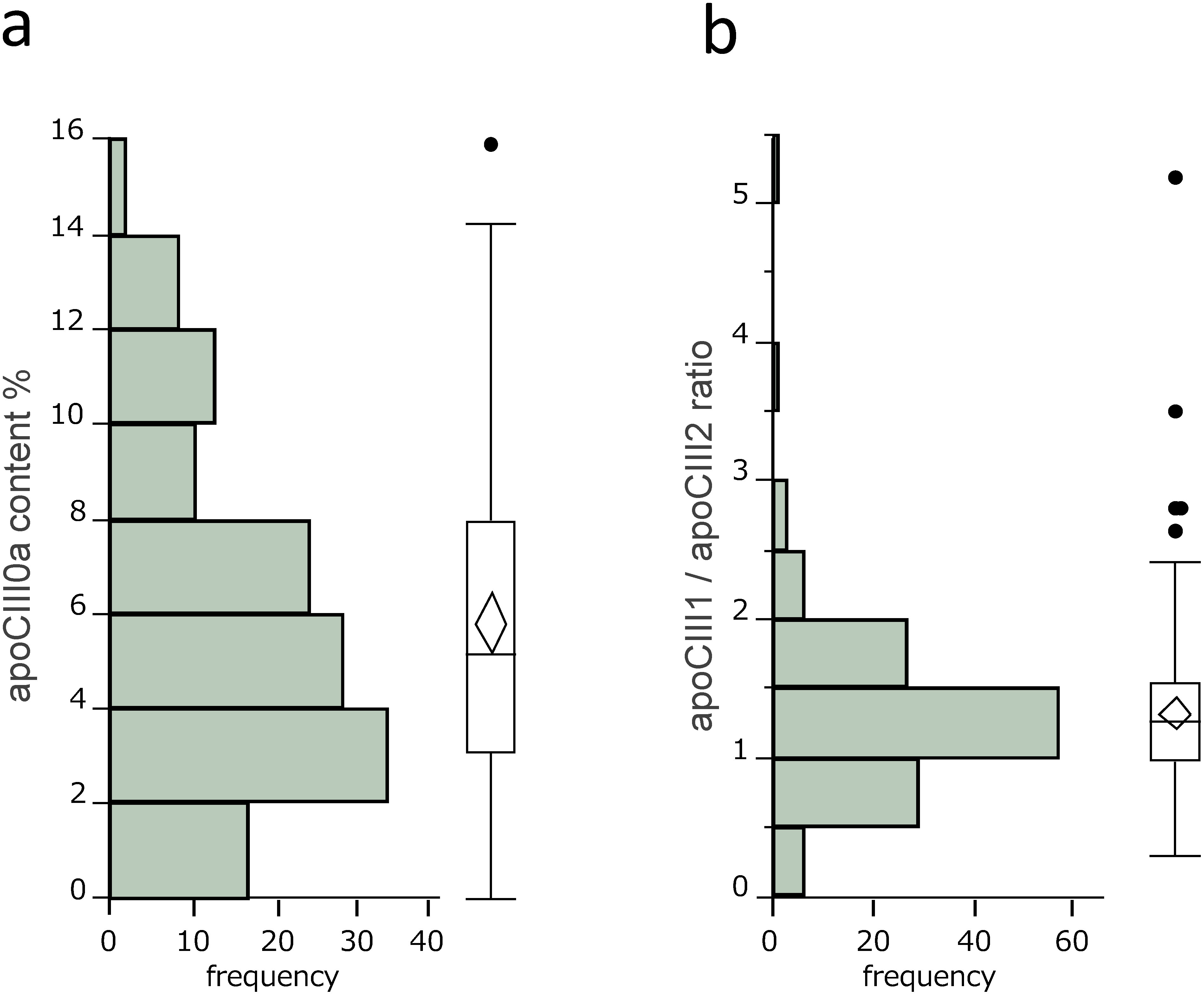
Fig. 6. Distribution of apoCIII0a content and apoCIII1/apoCIII2 ratio (*n*=130). The apoCIII0a content % (a) and apoCIII1/apoCIII2 ratio (b) represent site occupancy and sialylation, respectively. The upper limit of the reference range calculated using 90% sample quantiles was 11.5% and 1.92 for apoCIII0a content and apoCIII1/apoCIII2 ratio, respectively.

The distribution of the biomarkers in a cohort of 130 individuals (mean 4.8 years) is summarized in Fig. 6. The interquartile range of the apoCIII0a content was 3.0–8.0% and the upper limit of the reference range was 11.5% when calculated using 90% sample quantiles, and that of the apoCIII1/apoCIII2 ratio was 0.96–1.55, with an upper limit of 1.92.

**Figure 7:**
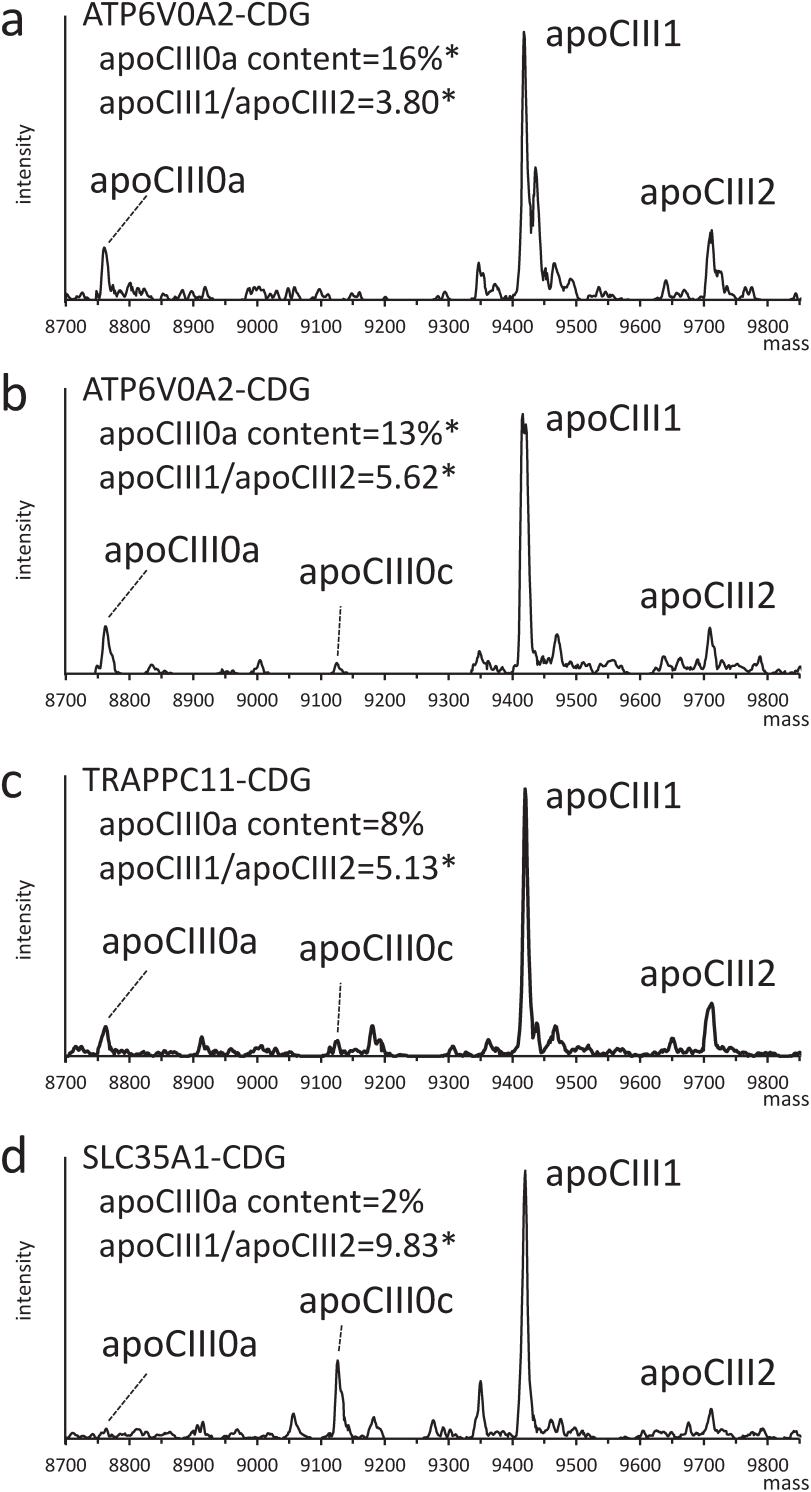
Fig. 7. Deconvoluted mass spectra of apoCIII from CDG patients with abnormal *O*-glycosylation. (a, b) ATP6V0A2-CDG, (c) TRAPPC11-CDG, (d) SLC35A1-CDG. All of these patients had reduced sialylation, and the ATP6V0A2-CDG patients showed an increase in apoCIII0a content. *significant. The spectrum data files are available in J-STAGE Data. https://doi.org/10.50893/data.massspectrometry.20076452

Finally, four CDG cases were analyzed (Fig. 7). ATP6V0A2-CDG, or autosomal recessive cutis laxa type II, was the first disease reported to be defective in both *N*- and *O*-glycans.^27)^ The abnormal glycosylation of ATP6V0A2-CDG is caused by an impairment in Golgi trafficking and function,^28)^ and sialylation is particularly affected. In some cases, a reduced site occupancy of *O*-glycan was reported as well.^13)^ In the cases presented in Figs. 7a and 7b, the apoCIII0a/apoCIII2 ratio and apoCIII0a contents were significantly elevated. Mutations in *TRAPPC11*, a subunit of the TRAPPIII complex, delay vesicular transport in the Golgi apparatus,^29)^ causing a decreased sialylation of both *N*- and *O*-glycans. As shown in Fig. 7c, the apoCIII1/apoCIII2 ratio was high. A deficiency of the SLC35A1 CMP-sialic acid transporter directly affects the sialylation of both *N*- and *O*-glycans and causes a severe reduction of sialylation.^30)^ As shown in Fig. 7d, the level of apoCIII2 was severely decreased and apoCIII0c was detected.

## CONCLUSION

ESI MS of apoCIII requires a pre-analysis purification step, while MALDI MS does not. However, given the widespread use of ESI instruments in clinical laboratories, ESI MS is better suited to facilitate the diagnosis of *O*-glycosylation disorders. Two diagnostic biomarkers, *i.e.*, reduced site occupancy and reduced sialylation, were assessed by the apoCIII0a content and the apoCIII1/apoCIII2 ratios, respectively. The use of DP to increase the intensity of ions derived from the major apoCIII isoform can induce the fragmentation of *O*-glycans, especially the Thr–GalNAc linkage, and risks making a false positive for reduced site occupancy at a high DP.

## Data Availability

The spectrum data files of Figs. 2, 3, 5, 7 and Supplementary Figure S2 are available in J-STAGE Data.

## Notes

Mass Spectrom (Tokyo) 2022; 11(1): A0104
