# Supplementary material for: Electrospray Ionization Mass Spectrometry of Apolipoprotein CIII to Evaluate O-glycan Site Occupancy and Sialylation in Congenital Disorders of Glycosylation
Source: Mass Spectrom (Tokyo). 2022 Aug 10;11(1):A0104. doi: 10.5702/massspectrometry.A0104 (PMC9396207; doi:10.5702/massspectrometry.A0104)
Supplement: Supplementary Data [file massspectrometry-11-1-A0104_s001.pdf]

Supplementary Figure S1

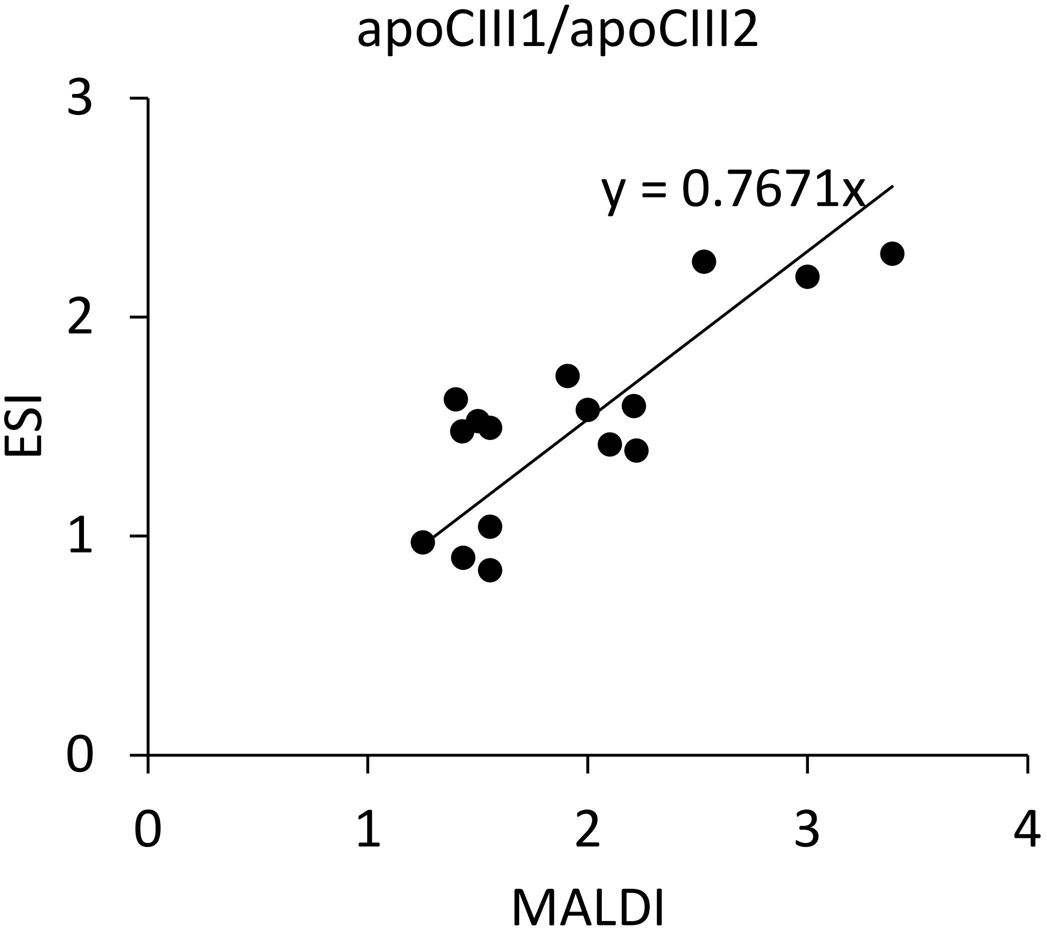

ApoCIII1/apoCIII2 ratios from ESI and MALDI mass spectra.
Same samples (n = 16) were analyzed by ESI MS and MALDI MS and the ratios of apoCIII1 and 2 ion intensity were plotted for comparison of sialic acid loss. MALDI MS was performed according to our previous report (J Mass Spectrom. 2021; 56(4):e4597. doi: 10.1002/jms.4597).

Supplementary Figure S2

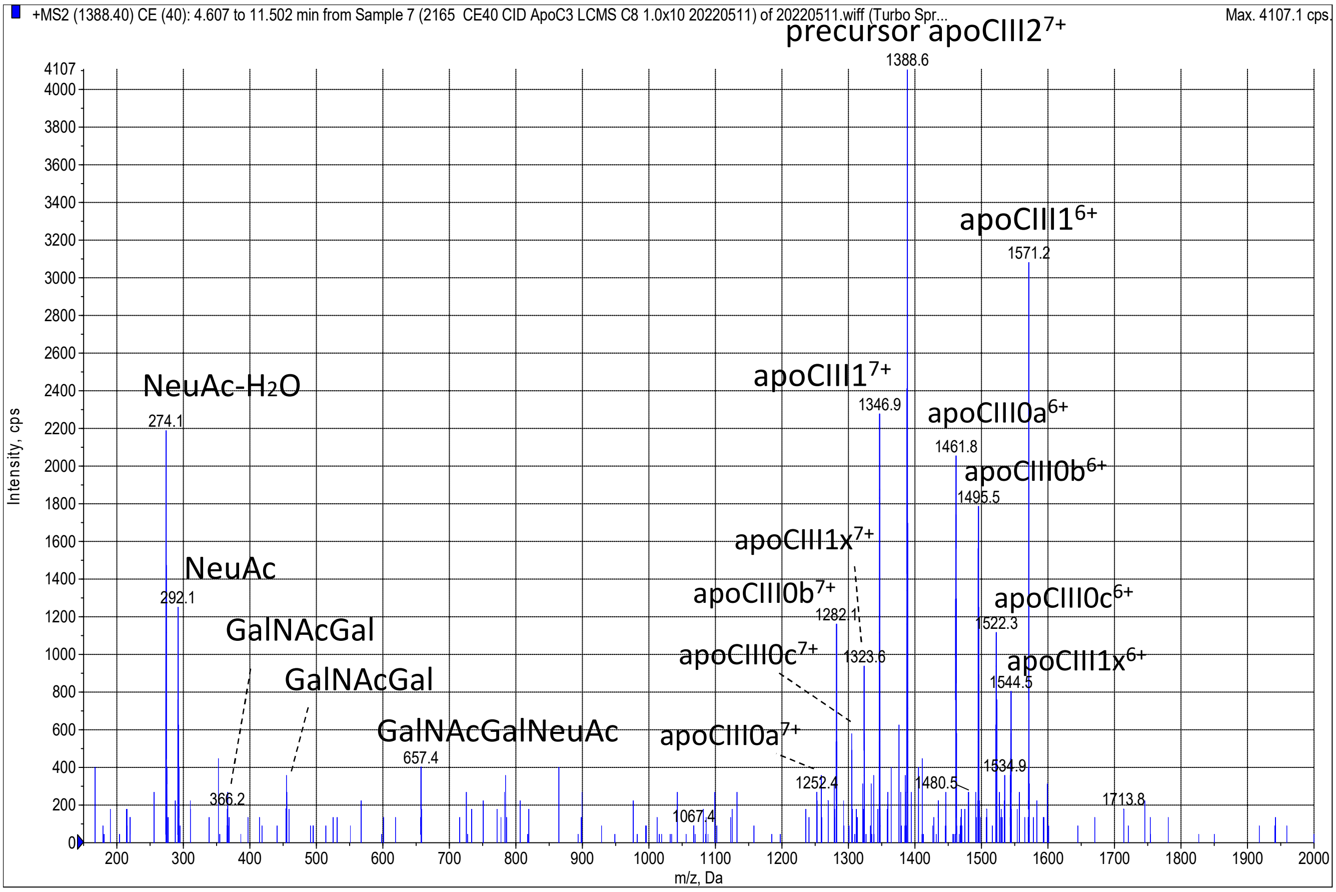

MS/MS spectrum of [apoCIII2+7H]$^{7+}$ ion activated by 40 V collision energy

The spectrum data files are available in J-STAGE Data.

https://doi.org/10.50893/data.massspectrometry.20076473
